# Tuberculosis in Otorhinolaryngology: Clinical Presentation and Diagnostic Challenges

**DOI:** 10.1155/2011/686894

**Published:** 2011-10-25

**Authors:** Rajiv C. Michael, Joy S. Michael

**Affiliations:** ^1^Department of ENT, Christian Medical College, Vellore 632004, Tamil Nadu, India; ^2^Department of Microbiology, Christian Medical College, Vellore 632004, Tamil Nadu, India

## Abstract

Tuberculosis affects all tissues of the body, although some more commonly than the others. Pulmonary tuberculosis is the most common type of tuberculosis accounting for approximately 80% of the tuberculosis cases. Tuberculosis of the otorhinolaryngeal region is one of the rarer forms of extrapulmonary tuberculosis but still poses a significant clinical and diagnostic challenge. Over three years, only five out of 121 patients suspected to have tuberculosis of the otorhinolaryngeal region (cervical adenitis excluded) had *Mycobacterium tuberculosis* culture-proven disease. Additional 7 had histology-proven tuberculosis. Only one patient had concomitant sputum-positive pulmonary tuberculosis. We look at the various clinical and laboratory aspects of tuberculosis of the otorhinolaryngeal region that would help to diagnose this uncommon but important form of extrapulmonary tuberculosis.

## 1. Introduction

 Though *Mycobacterium tuberculosis *infection can occur in all tissues of the body, pulmonary tuberculosis infection is overwhelmingly the most common type of infection representing approximately 80% of all cases of tuberculosis (TB) [1]. Among the extrapulmonary tuberculoses, the most common manifestation is lymphadenitis [2]. Tuberculosis of otorhinolaryngeal region is an uncommon, but not a rare, clinical problem. The commonest otorhinolaryngeal manifestation of TB is laryngeal tuberculosis excluding cervical lymphadenitis [3]. Previous reports state that around 25–30% of patients with otorhinolaryngeal TB have concomitant pulmonary TB [4]. However, since 1990, there have been reports of cases without pulmonary tuberculosis infection. Tuberculosis of the mastoid air cells and the middle ear is the next commonest manifestation in this group. Symptoms and signs of tuberculosis of this region can mimic malignancy, and; hence, an early diagnosis is essential. 

 Most physicians do not consider TB in the differential diagnosis of various otorhinolaryngeal symptoms, resulting in misdiagnosis and improper treatment. In addition, AIDS and other immunosuppressive diseases or treatments have increased the incidence and spectrum of tuberculosis [2]. 

 The diagnosis of TB is mainly based on a positive mycobacterial smear and culture or the histopathological presence of a chronic/caseating granuloma. Since there are a reasonable number of differential diagnoses to a clinical presentation of tuberculosis of the otorhinolaryngeal region, we report our clinical and laboratory experience with the clinical presentation, diagnosis, and treatment of tuberculosis of the otorhinolaryngeal region.

## 2. Materials and Methods

 This was a retrospective analysis of the samples received from the otorhinolaryngeal region, by the Mycobacteriology Laboratory of the Microbiology Department from 2007 to 2009 from the Otorhinolaryngology Department at the Christian Medical College, Vellore. This is a large tertiary care teaching hospital in Tamil Nadu, South India. The Mycobacteriology Laboratory is accredited to be a culture and drug susceptibility testing (DST) laboratory by the Revised National Tuberculosis Control Program (RNTCP), the Government of India and the Central tuberculosis division (CTD), New Delhi. 

 Patients with signs and symptoms suggestive of TB of the otorhinolaryngeal region underwent thorough clinical examination at the outpatient clinic of the Otorhinolaryngology Department at the Christian Medical College and Hospital. These patients underwent procedures to obtain biopsy for histopathological and or microbiological diagnosis. Blood investigations such as total and differential white blood cell count and ESR were done to assess the general condition of the patient. Patients with cervical adenitis were not included in this study.

### 2.1. Clinical Presentation

 A diagnosis of tuberculosis was based on the following clinical features.

#### 2.1.1. Laryngeal Tuberculosis

 The presenting symptoms are typically hoarseness, odynophagia, and dysphagia along with loss of weight and loss of appetite. Indirect laryngoscopy/fibroptic laryngoscopic examination commonly reveals diffuse erythema and granulomatous or polypoidal changes of the vocal cords. The classical clinical features of laryngeal tuberculosis are, however, seldom seen in modern clinical practice, and a biopsy is essential to establish the diagnosis and rule out a malignancy. The patients suspected to have TB were subjected to a microlaryngoscopy and biopsy under general anesthesia. Specimens are sent for histopathology examination, mycobacterial culture, and susceptibility testing.

#### 2.1.2. Tuberculosis of the Middle Ear

 The presenting symptoms are typically otorrhea which is persistent despite multiple courses of antibiotics, otalgia, hearing loss, and, in extreme cases, facial palsy. Physical examination findings include abundant polypoid or avascular pale granulation tissue. The patients with exuberant pale middle ear and mastoid granulations underwent a mastoid exploration or cortical mastoidectomy, and these specimens are sent for histopathology examination and mycobacterial culture and susceptibility testing.

#### 2.1.3. Tuberculosis of the Nasal and the Paranasal Region

 Nasal obstruction and blood-stained rhinorrhoea are the most common symptoms with which these patient present. It may be associated with frank epistaxis and headache. On examination of these patients, those with granular nasal and nasopharyngeal lesions underwent a rigid nasal endoscopy and biopsy of the lesion. Specimens are sent for both histopathology examination and mycobacterial culture and susceptibility testing.

### 2.2. Laboratory Diagnosis

 Tissue samples were sent for laboratory investigations to both the Histopathology Laboratory and the Microbiology Laboratory for mycobacterial smear, culture, and DST. Concurrent pulmonary tuberculosis was ruled out by sending 3 consecutive sputum samples for mycobacterial smear and culture. 

 Histopathological examination was done by staining the tissue sections with haematoxylin-eosin stains. The presence of chronic granulomatous inflammatory exudates, with or without caseation, was pathognomonic of tuberculosis. 

 Tissue samples sent to the Mycobacteriology Section were ground with a sterile homogenizer. Smear microscopy of the ground material was done by fluorescence auramine “O” method and graded as per the WHO/RNTCP method [5]. The material was digested and decontaminated by the modified Petroff's method and inoculated into Lowenstein-Jensen (LJ) media [6]. Drug susceptibility testing was done on *M. tuberculosis* isolates by 1% proportion method on LJ against Isoniazid, Rifampicin, Streptomycin, and Ethambutol [6].

## 3. Results

 A total of 121 samples were received from the Otorhinolaryngology Department to the Microbiology Department for mycobacterial culture and susceptibility testing over a period of 3 years from 2007 to 2009. These tissue specimens were also sent to the Pathology Department for histopathological examination. 

 As shown in Table 1, we received samples from patients with suspected TB from all three regions, namely, nasopharynx (18/36), larynx (13/33), and middle ear/mastoid cavity (46/51). Among the 121 patients, five had histopathological and culture-proven Tb—one each from the nasopharynx and larynx and three from the middle ear cleft. Seven patients had only histopathological appearances of chronic granulomatous inflammation strongly suggestive of tuberculosis. Among the remaining patients, 77 had non-specific inflammatory appearance on histology and were culture negative for tuberculosis. 

 The details of the five patients with *M. tuberculosis* culture positive and the histology suggestive of tuberculosis are given on Table 2. Patients were all adults with age ranging from 22 to 68 years and preponderance of males (4 : 1). Erythrocyte sedimentation rate (ESR) is an indirect indicator of inflammation and is used as a marker for tuberculosis. These patients had ESR ranging from 15 mm to 45 mm/hour. None of these five patients had coexistent pulmonary tuberculosis. 

Table 3 gives the details of the patients with a histopathological picture strongly suggestive of tuberculosis, but mycobacterial smear and culture of the tissue were negative. Here, only one patient with laryngeal tuberculosis had coincidental sputum smear-positive pulmonary tuberculosis. The ESR ranged from 5 mm to 50 mm.

## 4. Discussion

 Tuberculosis (TB) is one of the most common granulomatous infections that involve the otorhinolaryngeal region. With the advent of antituberculosis chemotherapy, the incidence has come down significantly, but there is a resurgence of extrapulmonary TB (EPTB) including primary otorhinolaryngeal TB due to human immunodeficiency virus (HIV). 

In our series over a period of 3 years, 12 of 121 patients had TB with only 5 being culture and histopathology proven and the remaining 7 with only histopathology strongly suggestive of tuberculosis. Only 1 patient had concomitant pulmonary TB. This highlights the difficulty in diagnosing laboratory-proven cases of EPTB. Moreover, clinical diagnosis of TB of the otorhinolaryngeal region is also challenging as the symptoms may mimic many other infective and noninfective pathological conditions. Laryngeal tuberculosis must be differentiated from other chronic infections such as syphilis, leprosy, fungal infections, and noninfective conditions like neoplasm, Wegener's granulomatosis, and sarcoidosis. This is possible through a histopathological examination since each of these conditions has a characteristic picture on histology. 

 Some forms of otorhinolaryngeal tuberculosis especially laryngeal and middle ear tuberculosis have been historically associated with coincidental pulmonary tuberculosis [7]. However, our experience has been similar to more recent reports, where only one of our patients with histopathology-proven laryngeal tuberculosis had coexistent pulmonary tuberculosis. Majority of our patients had primary otorhinolaryngeal tuberculosis. 

 HIV-infected persons are at markedly increased risk for primary or reactivation of TB especially extrapulmonary TB [8]. Studies have shown an increased risk of otorhinolaryngeal TB in patients with HIV [9], but, in our series, none of our patients with culture- or histopathologically-proven otorhinolaryngeal TB had HIV infection. 

 ESR values >10 mm have been associated with TB. Our patients with culture and or histopathological proven forms of otorhinolaryngeal tuberculosis had a mean ESR of 20 cu mm/hour. Erythrocyte sedimentation rate (ESR) is a nonspecific inflammatory marker that is commonly used during the initial diagnostic workup of patients with TB [10]. 

 In the Microbiology Laboratory, smear microscopy to detect acid fast bacilli and culture are used for diagnosis. Since these samples are paucibacillary in nature, they are often AFB smear negative. Although the organism can take 6 weeks or longer to grow on solid culture media (e.g., the egg-based Lowenstein-Jensen medium or the agar-based middle brook 7H10 or 7H11), growth generally occurs within 7–21 days with liquid culture media. Culture is also necessary to perform drug susceptibility testing. On the other hand, molecular techniques, such as polymerase chain reaction, detect DNA or RNA from samples with much faster results than culture and can often be obtained in 24–48 hours [11]. 

In our series, all of the 12 patients with either *M. tuberculosis *culture-proven or histopathology suggestive of TB were referred to their respective directly observed treatment short-course (DOTS) clinics and treated according to the Revised National Tuberculosis Programme (RNTCP) Guidelines on category I treatment with 4 drugs [12] intensive phase for 2 months with Isoniazid, Rifampicin, Pyrazinamide, and Ethambutol followed by 4 months of continuation phase with Isoniazid and Rifampicin. Multidrug-resistant tuberculosis (MDR-TB) is an increasing problem in patients with pulmonary tuberculosis in our country. So far, all the patients in our series have been infected with drug-susceptible *M. tuberculosis*. Antituberculous chemotherapy remains the cornerstone of treatment for extrapulmonary tuberculosis, and the role of surgery is mainly to establish an early diagnosis and initiate early treatment. 

 In conclusion, although the otorhinolaryngeal manifestations of tuberculosis are less common than those in the past, a high index of suspicion is necessary given the similarity in clinical presentation and appearance particularly to head and neck malignancies and other chronic noninfective and infective pathological conditions. In addition, these extrapulmonary extrascrofula lesions of the otorhinolaryngeal subsites are usually paucibacillary. Primary Otorhinolaryngeal tuberculosis may present without coexistent pulmonary tuberculosis. A positive mycobacterial culture along with a typical histopathological appearance remain the cornerstone of diagnosis. Thus it is our recommendation that all specimens from suspected cases of otorhinolaryngeal tuberculosis should have representative biopsies sent for histopathological examination as well as mycobacterial culture and sensitivity. Studies are required to look at the role of newer and more rapid laboratory methods for an earlier and a more accurate diagnosis of extrapulmonary tuberculosis.

## Figures and Tables

**Table 1:** Summary of the specimens received in the Mycobacteriology Section over 3 years with a suspicion of otorhinolaryngeal tuberculosis.

	Nasopharynx	Larynx/vocal cord	Middle ear/mastoid antrum
Tuberculosis culture & histopathology proven	1	1	3
Tuberculosis histopathology suggestive only	2	4	1
Nonspecific inflammation on histology	18	13	45
Fungal infection	9	3	1
Carcinoma	6	12	1

Total	36	33	51

**Table 2:** Details of five patients with *Mycobacterium tuberculosis* culture-positive otorhinolaryngeal specimens.

No.	Age	Sex	Specimen	HIV	ESR (mm)	Tissue specimen from affected site			Corresponding sputum AFB smear
						AFB smear	Mycobacterial culture	Histopathology	
1	22	M	Mastoid antrum	Negtive	45	Negtive	Scanty *M. tuberculosis *	Granulomatous inflammation	Negative
2	25	M	Mastoid antrum	Negtive	15	3+ AFB	Heavy *M. tuberculosis *	Granulomatous inflammation	Negative
3	31	M	Mastoid antrum	Negtive	25	Negtive	Scanty *M. tuberculosis *	Granulomatous inflammation	Negative
4	68	M	Larynx	Negtive	18	Negtive	Scanty *M. tuberculosis *	Granulomatous inflammation	Negative
5	24	F	Nasopharynx	Negtive	21	Negtive	Scanty *M. tuberculosis *	Granulomatous inflammation	Negative

**Table 3:** Details of seven patients with histopathology-positive and mycobacterial culture-negative otorhinolaryngeal specimens.

No.	Age	Sex	HIV	ESR (mm)	Specimens	Tissue specimen from affected site			Corresponding Sputum AFB smear
						AFB smear	Culture	Histopathology	
1	38	M	Negative	5	supraglottis	Negative	No growth	Granulomatous inflammation suggestive of TB	1+ AFB
2	16	F	Negative	10	Soft palate	Negative	No growth	Granulomatous inflammation suggestive of TB	Negative
3	21	F	Negative	15	Mastoid antrum	Negative	No growth	Necrotising granuloma suggestive of TB	Negative
4	55	F	Negative	50	Nasopharynx	Negative	No growth	Granulomatous inflammation suggestive of TB	Negative
5	22	F	Negative	15	Supraglottis	Negative	No growth	Necrotising granuloma suggestive of TB	Negative
6	54	M	Negative	15	Larynx	Negative	No growth	Granulomatous inflammation suggestive of TB	Negative
7	62	M	Negative	7	Larynx	Negative	No growth	Caseating granulomatous inflammation suggestive of TB	Negative
